# Cordarone Extravasation Inducing Volkmann’s-like Syndrome

**DOI:** 10.4021/cr120w

**Published:** 2011-11-20

**Authors:** Paolo Simoni, Laura Scarciolla, Carole Maréchal, Selma Ben Mustapha, Bruno Beomonte Zobel

**Affiliations:** a Service of MSK imaging, University Hospital of Liege (CHU), Domain du Sart Tilman Bat. 45, 4000-B Liege, Belgium; b Departement of Radiology, University Campus Bio-medico, Via Alvaro del Portillo, 200, 00128 Rome, Italy; c Service of Anesthesiology, University Hospital of Liege (CHU), Domain du Sart Tilman Bat. 45, 4000-B Liege, Belgium

**Keywords:** Amiodarone, Adverse effects, Skin pathology, Necrosis chemically induced

## Abstract

We report a case of Volkmann’s-like syndrome occurred after an extravasation of 300 mg of cordarone administrated for a cardiac arrest.The day following the extravasation, an extensive necrosis of the skin and soft tissues occurred. The patient progressively developed a retraction of the muscles of her forearm. To the best of our knowledge this is first the reported case of an acute necrosis of the soft tissue inducing a Volkmann’s-like retraction of the upper limb subsequent to a cordarone extravasation. The imaging findings are provided along with a review of the literature.

## Introduction

We describe a Volkmann’s-like syndrome occurred after an extravasation of cordarone at the intensive care unit. Volkmann’s syndrome usually develops after a supracondylar fracture especially when constricting bandages or plaster casts obstruct circulation, thus reducing blood supply. We herein illustrate a case of a muscular fibrosis and contracture mimicking a classic Volkmann’s syndrome, due to the direct toxic effect of cordarone.

## Case Report

A 44-year-old woman with a severe pneumonia was treated by a bolus of 300 mg of cordarone administrated at the intensive care unit for a cardiac arrest after a ventricular fibrillation. A large extravasation of cordarone accidentally occurred, causing a mild swelling of her left forearm. The day following the extravasation, the patient developed a large area of skin changes with dark discoloration, oedema and some tense blisters at the posterior aspect of her left forearm.

**Figure 1:**
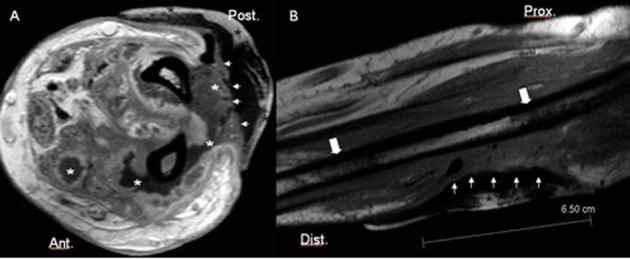
Axial T1-weighted images after injection of Gadolinium (A) and sagittal T1-weighted images before contrast injection (B). Multiple confluent avascular areas of soft tissues necrosis (white asterisks) of the posterior and the anterior compartment of the forearm are observed. The large area of skin necrosis (white arrowheads) is covered with a bandage (black asterisk). Some areas of bone marrow oedema of the radius are seen near the abnormal changes of soft tissue (large white arrows).

The patient was treated with cold packs, local disinfection, topical steroid cream. Oral therapy by amoxicillin for pneumonia was continued at dose of 500 mg per day. Withnotstanding the local treatment, forty-eight hours after the extravasation of cordarone, a large area of skin necrosis rapidly developed at the cubital aspect of the forearm. A Magnetic Resonance Imaging (MRI) (Fig.1) with contrast injection was performed in order to assess deep soft tissues and bone marrow involvement.

**Figure 2:**
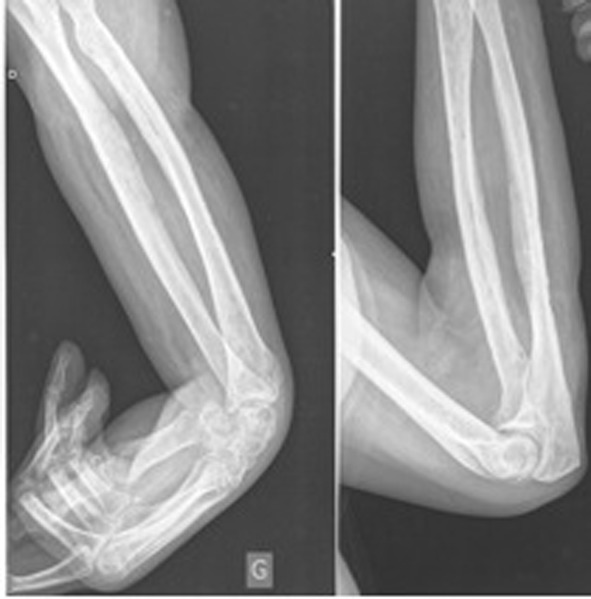
X-rays performed 12 months after cordarone extravasation show a severe contracture of the upper limb involving elbow, wrist and interphalangeal joints of the fingers. Notice the atrophy of the soft tissues of the forearm (reduced diameter of the forearm and hypo-density due to the fatty-fibrous atrophy).

MRI showed multiple confluent avascular areas of soft tissues necrosis of the posterior and anterior compartment of the forearm. Some areas of abnormal signal intensity of the bone marrow were also detected at level of radius and ulna. Patient was treated with a large fasciotomy and a soft tis¬sue debridement. No nerve damage was observed at surgery. Microbiological analysis of the necrotic tissue removed at surgery revealed no tissue infection and the extensive necrosis of soft tissue were therefore linked to the acute toxic effect of cordarone. After surgical treatment the patient progres¬sively developed a deformity of her upper limb including el¬bow flexion, forearm pronation, flexion-adduction of wrist and thumb, digital metacarpo-phalangeal joint extension, and inter-phalangeal joint flexion, mimicking the classical sequelae of a Volkmann’s disease (Fig.2).

## Discussion

Volkmann’s syndrome is an ischemic contracture of the muscles followed by fibrosis and upper arm retraction and deformity, first described by Richard von Volkmann in 1881. This deformity usually occurs in children after a supracondylar fracture of the humerus [1]. The acute compression of the brachial artery caused by the fracture and subsequent hematoma may induce an acute ischemia, necrosis and fibrotic retraction of the flexor muscles of the forearm, especially of the flexor digitorum profondus and flexor pollicis longus muscles [1]. The typical deformities include elbow flexion, forearm pronation, flexion and adduction of thumb and wrist, digital metacarpo-phalangeal joint extension and inter-phalangeal joint flexion [1]. The risk of developing a Volkmann’s syndrome increased by constricting bandages or plaster casts applied. Other very rare causes of upper limb deformity mimicking a Volkmann disease have been reported. Newmeyer WL et al observed Volkmann- like deformity of the upper limb after drugs overdose, injury of main arterial trunks, and in patients affected by myelogenous leukemia [2].

In our case, the patient presented with an area of skin necrosis on the left forearm and a contracture in flexion of the wrist and the elbow after an extravasation of cordarone. Extensive skin necrosis and soft tissue necrosis of the forearm has been reported after large coradone extravasation [3]. The Committee for the Safety of Medicines has only two entries for amiodarone extravasation injury and nine for injection site reactions [3]. However, extravasation is not un¬common and care should be taken to avoid severe reaction of skin and soft tissues. It has been speculated that toxic effects of the amiodarone infusion are due to the low pH of the solution (between pH 3.5 and 4.5) and the additives polysorbate and benzyl alcohol [3]. In our case the acute toxic effect of cordarone caused an extensive necrosis of the skin nearby the site of extravasation as well as an extensive necrosis of the soft tissue of the anterior and posterior forearm and associated with bone marrow edema. A suprainfection of skin in correspondence of the area necrosis was ruled out by microbiological tests on necrotic tissue removed at surgery. In addition there was no evidence of compartment syndrome.

To the best of our knowledge this is the reported case of an acute necrosis of the soft tissue inducing a Volkmann’s-like retraction due to a cordarone extravasation. Upper limb retraction may be treated either with a non-operative approach, as in our case, consisting in a comprehensive rehabilitation program or with surgical tendon lengthening and skin grafting [4, 5]
